# Supplementary material for: Rapid Monitoring of Viable Genetically Modified Escherichia coli Using a Cell-Direct Quantitative PCR Method Combined with Propidium Monoazide Treatment
Source: Microorganisms. 2023 Apr 26;11(5):1128. doi: 10.3390/microorganisms11051128 (PMC10220751; doi:10.3390/microorganisms11051128)
Supplement: Supplementary file 1 [file microorganisms-11-01128-s001.zip › microorganisms-2337529-supplementary.pdf]

Table S1. Repeatability and precision of dual-plex qPCR assay for antibiotic resistant genes and taxon-specific genes using serial dilutions of plasmid and genome DNA from two genetically modified *Escherichia coli* (*E.coli*) strains.

Strains	Targets	True DNA copy	Mean Cq	Mean DNA copy	SD	RSDr %	Bias %	Total DNAs (ng)
pJ281 (Plasmid insertion)	*KmR* (Single-plex)	100000000	15.31	84518734	0.05	0.35	-15.48	0.842
		10000000	18.25	12411955	0.13	0.70	24.12	0.0842
		1000000	21.86	1181000	0.20	0.92	18.10	0.00842
		100000	25.94	82138	0.12	0.47	-17.86	0.000842
		10000	29.36	8783	0.12	0.41	-12.17	0.0000842
		1000	32.52	1119	0.37	1.14	11.86	0.00000842
	dxs (Dual-plex)	100000000	15.84	80220382	0.08	0.58	-19.78	500
		10000000	18.69	12134034	0.09	0.54	21.34	50
		1000000	22.36	1066923	0.08	0.38	6.69	5
		100000	25.94	99389	0.09	0.38	-0.61	0.5
		10000	29.18	11565	0.37	1.39	15.65	0.05
		1000	33.14	838	0.05	0.17	-16.25	0.005
BW25113 (Genome insertion)	*nptII* (Dual-plex)	100000000	15.48	82806062	0.12	0.75	-17.19	500
		10000000	18.43	11623493	0.11	0.61	16.23	50
		1000000	21.95	1118459	0.09	0.40	11.85	5
		100000	25.60	98926	0.19	0.73	-1.07	0.5
		10000	29.00	10330	0.06	0.22	3.30	0.05
		1000	32.65	911	0.42	1.27	-8.92	0.005
	dxs (Dual-plex)	100000000	15.50	81514281	0.05	0.31	-20.00	500
		10000000	18.51	11838679	0.15	0.81	16.42	50
		1000000	22.17	1131299	0.15	0.69	11.52	5
		100000	25.89	103641	0.12	0.45	2.42	0.5
		10000	29.32	11536	0.22	0.73	14.25	0.05
		1000	33.42	829	0.12	0.36	-17.69	0.005

(a) Specificity of dual-plex qPCR for pJ281 targeting *KmR/dxs* **(b)** Specificity of dual-plex qPCR for BW25113 targeting *nptII/dxs*

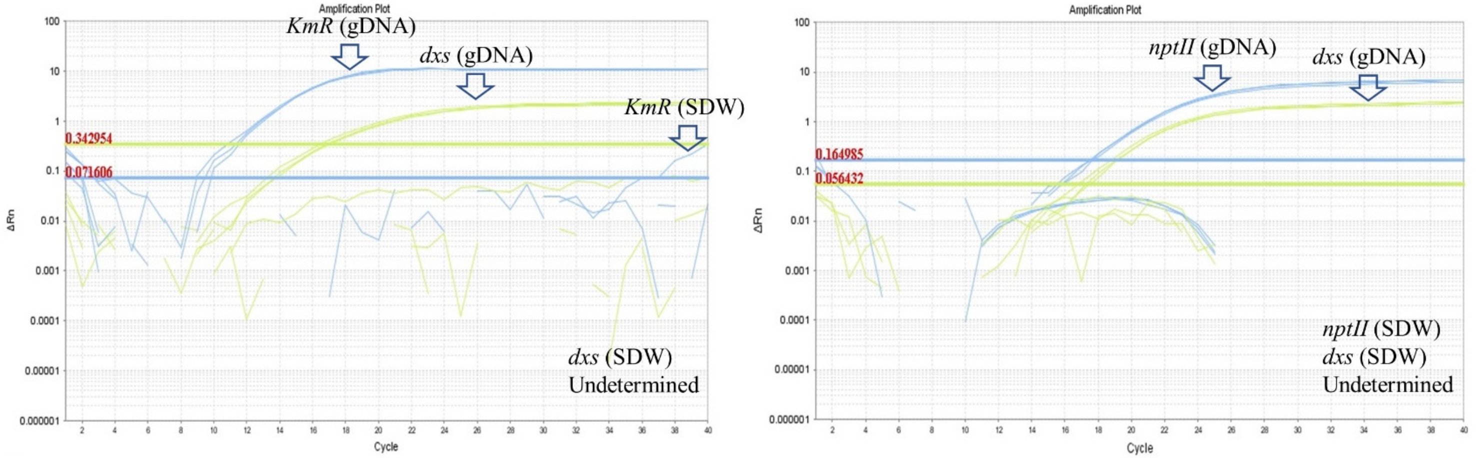

Figure S1. Dual-plex qPCR using genome DNA of two genetically modified *Escherichia coli* (*E.coli*) strains to verify specificity of primer and probe combinations. (a) qPCR for pJ281 targeting *KmR*/*dxs*; (b) qPCR for BW25113 targeting *nptII*/*dxs*; Sterile distilled water (SDW) was used as negative control.

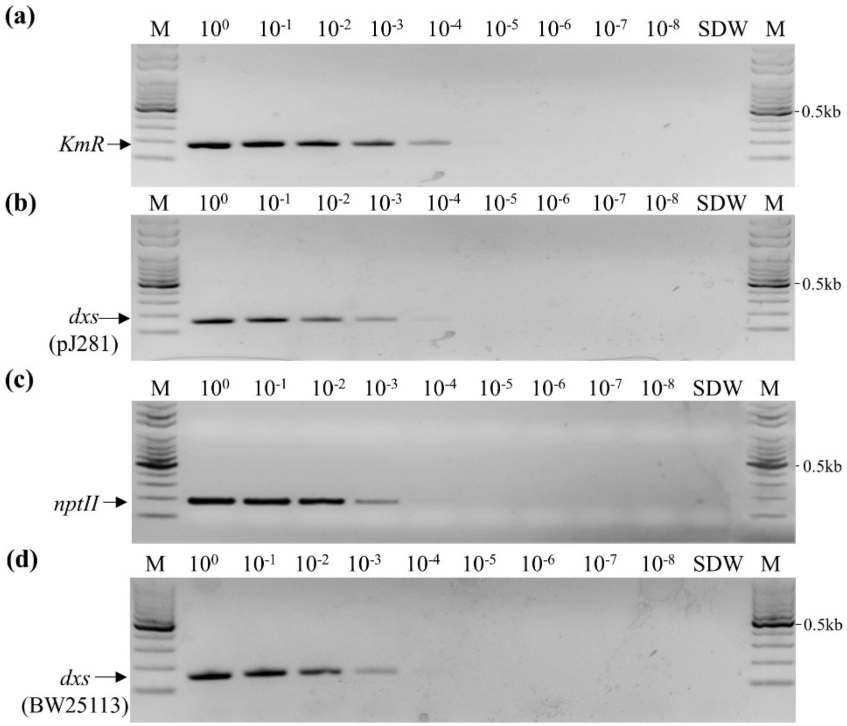

Figure S2. Primer specificity confirmation by **c**onventional PCR analysis using serially diluted cell suspension as template for two genetically modified *Escherichia coli* (*E.coli*) strains. 10^0 indicates overnight cell culture with a mean CFU of 2.39×10^6 per microliter (μL) for the strain pJ281 and 2.62×10^6 per microliter (μL) for the strain BW25113; SDW indicates nuclease-free water as the negative control.

(a) Limit of detection (LOD) for viable pJ281 cells by *KmR*/*dxs*

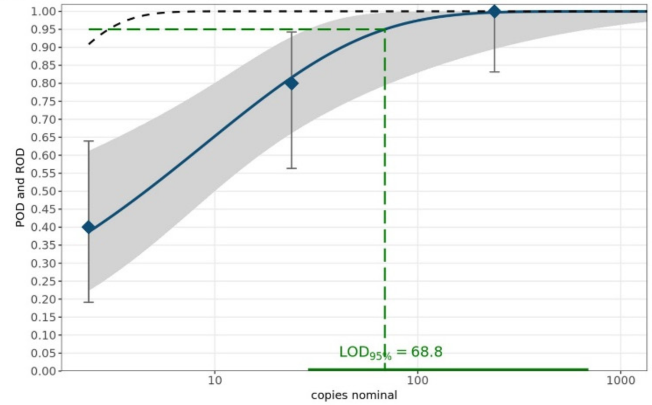

The plausibility check indicates that the value for the slope parameter b is significantly less than 1 (b = 0.54). This means the average amplification probability is higher at higher dilution levels than at lower dilution levels.
Such a situation can be related to: inhibitory matrix effects, a large variability in the amplification process from the one test to another under repeatability conditions, or accidental problems causing false positives if the number of copies of the target DNA sequence is less than 1.
POD curve and LOD95%
The LOD95% is 68.817 with a 95 % confidence interval of [28.806, 693.203].
The figure below summarises the results. The blue diamonds characterise the laboratory-specific RODs. The blue curve denotes the mean POD curve along with the corresponding 95 % confidence range highlighted as the grey band. The POD curve under ideal conditions is displayed as the black dashed curve.

(b) Limit of detection (LOD) for viable pJ281 cells by *nptII*/*dxs*

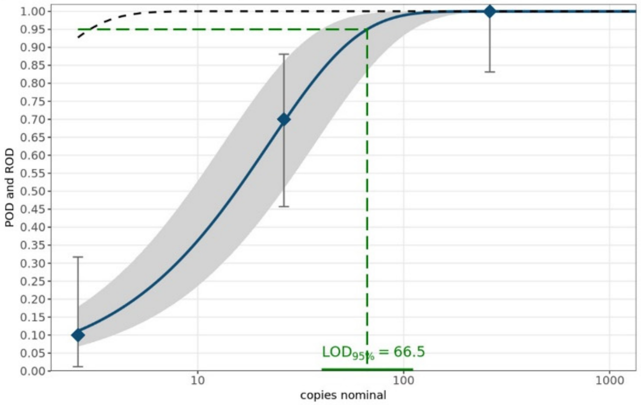

The plausibility check indicates no irregularities.
POD curve and LOD95%
The LOD95% is 66.469 with a 95 % confidence interval of [39.947, 111.155].
The figure below summarises the results. The blue diamonds characterise the laboratory-specific RODs. The blue curve denotes the mean POD curve along with the corresponding 95 % confidence range highlighted as the grey band. The POD curve under ideal conditions is displayed as the black dashed curve.

Figure S3. Plausibility check of limit of detection (LOD) at 95% confidence interval for viable *E.coli* cells harboring *KmR*- and *nptII*-resistant genes analyzed by Quodata web application.
